# Targeting the cancer glycocalyx in salivary duct carcinoma: tumor‐associated mucin 1 (Tn‐MUC1) as a novel cell surface marker

**DOI:** 10.1002/2056-4538.70042

**Published:** 2025-08-22

**Authors:** Masashi Kuroki, Ryo Kawaura, Hiroyuki Tomita, Hirofumi Shibata, Toshimitsu Ohashi, Tomohiko Ishikawa, Hideshi Okada, Akira Hara, Takenori Ogawa

**Affiliations:** ^1^ Department of Otolaryngology‐Head and Neck Surgery Gifu University Graduate School of Medicine Gifu Japan; ^2^ Department of Tumor Pathology Gifu University Graduate School of Medicine Gifu Japan; ^3^ Department of Head and Neck Surgery‐Otorhinolaryngology Ogaki Municipal Hospital Ogaki Japan; ^4^ Department of Otolaryngology‐Head and Neck Surgery Tohoku University Graduate School of Medicine Sendai Japan; ^5^ Department of Emergency and Disaster Medicine Gifu University Graduate School of Medicine Gifu Japan

**Keywords:** glycocalyx, glycan, lectin, Tn‐MUC1, salivary gland cancer, salivary duct carcinoma

## Abstract

Despite the use of targeted drugs for human epidermal growth factor receptor type 2 (HER2)‐positive salivary duct carcinoma (SDC) treatment, the overall prognosis for SDC remains poor. In this study, we aimed to investigate whether the glycocalyx of SDC cells serves as a potential cell surface marker. To understand the complex structure of the glycocalyx of salivary gland tumors, we used a lectin with glycan‐specific binding properties. Lectin staining was performed for five types of common salivary gland tumors. We determined the expression of the transmembrane glycoprotein mucin 1 with Tn antigen (GalNAc), Tn‐MUC1, using 20 clinical SDC specimens. *Vicia villosa* lectin (VVL) was not stained in the vascular endothelium but specifically stained in the SDC tumor cells. GalNAc, to which VVL binds, formed Tn‐MUC1 via glycosylation with N‐acetylgalactosaminyltransferases (GALNTs). GALNT7 was highly expressed in SDC. Analysis of clinical SDC specimens revealed that Tn‐MUC1 was also positive in the SDC tumor cells, suggesting its potential as a cell surface target for SDC.

## Introduction

Glycocalyx is a complex structure composed of mucopolysaccharides, such as glycoproteins, proteoglycans, and glycosaminoglycans. Both endothelial and tumor cells possess glycocalyx on their surface, and the glycocalyx structure was first observed via electron microscopy in the 1960s [1, 2]. Glycocalyx plays several roles in the vascular endothelium, including blood circulation, adhesion of inflammatory cells, and maintenance of homeostasis in vascular endothelial cells [3]. Cancer cells also have glycan in the form of glycocalyx and produce abundant glycocalyx, changing their phenotype due to the physical stress of their surroundings in the tumor microenvironment [4, 5]. Therefore, glycocalyx acts as a barrier and sensor on the surface of cancer cells that interacts with host bioactive substances, such as cytokines, and immune cells, such as lymphocytes [6].

Glycans are difficult to analyze owing to their complex structure and various components. Lectins that specifically bind to glycans are widely used to analyze their complex structure [7, 8]. Lectin staining is used to evaluate both the thickness of the glycocalyx on the cell surface and the specific composition and quantity of glycans in the glycocalyx [9]. Tissue fixation is crucial as glycans are unstable under chemical stimulation and hydration, and formalin‐fixed paraffin‐embedded (FFPE) tissues have lower lectin responsiveness than frozen specimens [10, 11].

In O‐glycosylation, a post‐translational modification, N‐acetylgalactosamine (GalNAc) is generally added to the protein transported to the Golgi apparatus via catalysis with N‐acetylgalactosaminyltransferases (GALNTs) [12]. GalNAc is added to serine/threonine residues via O‐glycosylation to form tumor‐associated mucin 1 (TA‐MUC1), which plays key roles in tumor invasion, metastasis, angiogenesis, proliferation, and apoptosis [13, 14, 15, 16]. GalNAc, to which *Vicia villosa* lectin (VVL) binds, forms Tn‐MUC1 via glycosylation. MUC1 in normal cells is modified with highly complex O‐glycans; however, in cancer cells, glycan elongation stops, resulting in the formation of abnormally short glycans and an increase in tumor‐associated glycans, such as Tn, T, STn, and ST antigens [17, 18]. Various diagnostic markers targeting these antigens, such as CA15‐3 for breast cancer, are currently used in clinical settings [19].

Salivary gland tumors are rare neoplasms of the head and neck, accounting for approximately 5% of all head and neck tumors. The overall incidence of salivary gland tumors is 7.03–8.58/100,000 [20], and the incidence of malignant tumors is even lower, 16/1,000,000 [21]. Salivary gland tumors are of various histological subtypes and are classified into 20 types of malignant tumor and 11 types of benign tumor according to the 5th edition of the World Health Organization (WHO) classification [22]. Based on histology, the incidence of malignant tumors is 20% for mucoepidermoid carcinoma (MEC), 12% for adenoid cystic carcinoma (ACC), and 10% for salivary duct carcinoma (SDC) [23]. In this study, we focused on two common benign and three malignant tumors with a particular focus on SDC. SDC is a high‐grade tumor with poor prognosis. For the treatment of salivary gland carcinoma, surgery is the first choice for resectable cases, and postoperative radiation therapy may also be required depending on the malignancy and histology. As salivary gland carcinomas have various histological subtypes and characteristics, a standard chemotherapy regimen has not yet been established. Recent studies have reported the effectiveness of molecular targeted drugs for human epidermal growth factor receptor type 2 (HER2)‐ and androgen receptor (AR)‐positive tumors [24, 25]. However, HER2 positivity in SDC is limited (30–40%) [24, 26, 27, 28] and the prognosis, especially for HER2‐negative SDC, is still poor. Salivary glands secrete mucins composed of glycoproteins; therefore, tumors that arise from the salivary glands are suitable for glycan analysis. However, there are no reports that comprehensively analyze glycans in salivary gland tumors or that verify the usefulness of tumor‐associated glycans. Here, we aimed to analyze the glycan structure in different histological types of salivary gland tumor, including two types of representative benign [pleomorphic adenoma (PA) and Warthin tumor (WT)] and three types of representative malignant (MEC, ACC, and SDC) tumors via lectin staining.

## Materials and methods

### Patients and sampling of fresh frozen specimens and FFPE material

This study was conducted in accordance with the Declaration of Helsinki and approved by the Ethics Committee of Gifu University (Gifu City, Japan). Informed patient consent and prior approval from the Gifu University Hospital affiliated with the Gifu University Graduate School of Medicine Ethics Committee (approval no. 2020‐234) were obtained before using the clinical materials in experiments. Informed patient consent was obtained before using the new clinical materials in experiments. If past clinical samples were used, informed consent was not sought because a waiver of consent was approved by the institutional review board due to the opt‐out approach.

The study population using fresh frozen specimens consisted of 12 patients with different salivary gland tumors (PA, *n* = 4; WT, *n* = 2; SDC, *n* = 2; MEC, *n* = 2; and ACC, *n* = 2) who underwent surgery at the Gifu University Hospital. The most common histological types of both benign and malignant tumors were selected. As glycan is water‐soluble and its structure is easily broken by formalin fixation, freshly frozen specimens were used in all experiments. Due to the lack of archived fresh‐frozen specimens from previous cases, new cases were prospectively collected, and this experiment was conducted as an initial screening. Immediately after excision, a sample of approximately 5 mm was collected, protected using Tissue OCT compound (Sakura Finetek, Japan), snap‐frozen in liquid nitrogen, and stored at −80 °C.

The study population using FFPE comprised 20 patients with SDC who underwent treatment at the Gifu University Hospital between May 2007 and September 2021. Regardless of clinical stage, only initial treatment cases were selected, and recurrent cases were excluded. Specimens from the initial surgery or initial biopsy were used.

All specimens were confirmed by two pathologists based on the 5th edition of the WHO Health Organization classification. All relevant clinical data were acquired from the patient medical records.

### Lectin histochemistry

In total, 20 types of lectins were used in this study. Screening kit I (Vector Laboratories, Burlingame, CA, USA) consisted of seven types of lectins: concanavalin A (Con A), *Dolichos biflorus* agglutinin (DBA), peanut agglutinin (PNA), *Ricinus communis* agglutinin (RCA) I, soybean agglutinin I (SBA), *Ulex europaeus* agglutinin (UEA) I, and wheat germ agglutinin (WGA). Screening kit II (Vector Laboratories) consisted of six types of lectins: *Griffonia simplicifolia* lectin (GSL) I, *Pisum sativum* agglutinin (PSA), *Len culinaris* agglutinin (LCA), *Phaseolus vulgaris* erythroagglutinin (PHA‐E), *Phaseolus vulgaris* leucoagglutinin (PHA‐L), and succinylated WGA. Screening kit III (Vector Laboratories) consisted of seven types of lectins: *Datura stramonium* lectin (DSL), *Erythrina cristagalli* lectin (ECL), GSL II, Jacalin, *Lycopersicon esulentum* lectin (LEL), *Solanum tuberosum* lectin (STL), and *Vicia villosa* lectin (VVL; supplementary material, Table S1). In this study, a comprehensive analysis of diverse glycan specificities was performed by utilizing all 20 lectins from Vector Laboratories' lectin screening kits I, II, and III. Previous research has validated the combined use of these kits as an effective approach for comprehensive glycan profiling in human and mammalian tissues [29, 30, 31].

First, 5‐μm sliced sections were fixed with 4% paraformaldehyde (FujiFilm, Tokyo, Japan) for 15 min. All sections were incubated in a 10% carbon‐free blocking solution (Vector Laboratories) for 1 h at approximately 20–25 °C to block non‐specific labeling. After washing with phosphate‐buffered saline (PBS, Wako, Osaka, Japan), sections were incubated with various lectins (0.5%) and 0.5% anti‐CD31 antibody (ab76533; dilution 1:200; Abcam, Cambridge, UK) to stain the vascular endothelial cells overnight at 4 °C. The following day, all sections were incubated with 0.5% Streptavidin DyLight®594 (SA‐5594‐1; dilution 1:200; Vector Laboratories) and 0.5% pre‐adsorbed Goat Anti‐Rabbit IgG H&L (DyLight®488; ab96899; dilution 1:200; Abcam) for 60 min at room temperature. After washing with PBS, all sections were incubated with 0.1% 4,6‐diamidino‐2‐phenylindole (DAPI, Dojindo, Kumamoto, Japan) solution for 5 min at room temperature.

### Fluorescence microscopy and image evaluation

Slides were evaluated using fluorescence microscopy and imaging software (CellSens, Olympus, Tokyo, Japan). Lectins, vascular endothelial cells, and cell nuclei were evaluated using GNA (red), fluorescein isothiocyanate (green), and DAPI (blue) staining, respectively. Images were taken at the same exposure time and labeled as ‘positive (+)’, ‘weakly positive (±)’, and ‘negative (−)’ according to the fluorescence intensity.

### RNA extraction and RT‐PCR

After cutting and macrodissecting 10‐μm sections, total RNA of SDC cells was extracted using the RNeasy Kit (QIAGEN, Venlo, the Netherlands). cDNA was synthesized using the SuperScript III First‐Strand Synthesis Kit (Life Technologies, Carlsbad, CA, USA). Reverse transcription‐polymerase chain reaction (RT‐qPCR) was performed using the StepOnePlus system (Applied Biosystems, Foster City, CA, USA). *GALNT* primers were designed using Primer‐BLAST (supplementary material, Table S2). The comparative Ct method was used to analyze the relative gene expression. RT‐qPCR was performed in triplicate for each primer. *Beta‐actin* was used as an internal control.

### Public RNA‐seq data

Here, mRNA expression datasets (GSE138581) for SDC were obtained from the National Center for Biotechnology Information database (https://www.ncbi.nlm.nih.gov/geo/). RNA‐seq data for *GALNT* was extracted and validated in SDC.

### Immunohistochemistry

Paraffin blocks from 20 SDC tumors were cut into 3‐μm thick sections and subjected to hematoxylin–eosin staining. Adjacent serial sections were subjected to immunohistochemistry for Tn‐MUC1 [anti‐human MUC1 therapeutic antibody (5E5); dilution 1:1000; Creative Biolabs, Shirley, NY, USA], VVL (Vector Laboratories, Newark, CA, USA), HER2 (A0485; dilution 1:800; Dako, Santa Clara, CA, USA), Ki‐67 (M7240; dilution 1:150; Dako), and GALNT7 (Catalog Number: 13962‐1‐AP, dilution 1:200; Proteintech, Rosemont, IL, USA). Negative controls were used to confirm that the reaction was not non‐specific.

Deparaffinized sections were subjected to autoclave boiling in 0.015 M sodium citrate buffer solution (pH 6.0, Sigma, St Louis, MO, USA) for 10 min at 110 °C for antigen retrieval before incubation with 3% H_2_O_2_ diluted in methanol for 10 min and blocked with 2% normal bovine serum (Sigma). For GALNT7 immunostaining, EDTA buffer, pH 8, was specifically used for antigen retrieval. Sections were incubated with primary antibodies overnight at 4 °C, followed by incubation with conjugated secondary antibodies for 60 min at 37 °C. The immunoreaction was visualized using 3,3'‐diaminobenzidine tetrahydrochloride (Sigma).

Tn‐MUC1 and VVL were quantified using ImageJ software (version: 2.1.0/1.53c, National Institutes of Health, Bethesda, MA, USA; 16‐bit, threshold; 150). In brief, using three micrographs of each case with a ×400 field of view, the threshold value of the staining intensity was set, and the average value of the positive area was evaluated. HER2 expression was determined to be negative (0, 1+) or positive (2+, 3+) according to the H score [32]. The number of Ki‐67‐positive cells was measured using a ×400 field of view. All staining evaluations were performed by one pathologist (HT) and one otolaryngologist (MK).

### Statistical analyses

Differences in categorical data between the two groups were analyzed using the chi‐square test. Correlations of staining area of VVL and TnMUC‐1 were examined using Pearson correlation coefficients. Survival time analysis was performed using the Kaplan–Meier method, and differences between groups were analyzed using the log‐rank test. All statistical analyses were conducted using the GraphPad Prism 8 software (GraphPad Software, Inc., San Diego, CA, USA). Statistical significance was set at *p* < 0.05.

## Results

### Representative glycans GalNAC and fucose are only expressed in malignant tumors

**Figure 1:**
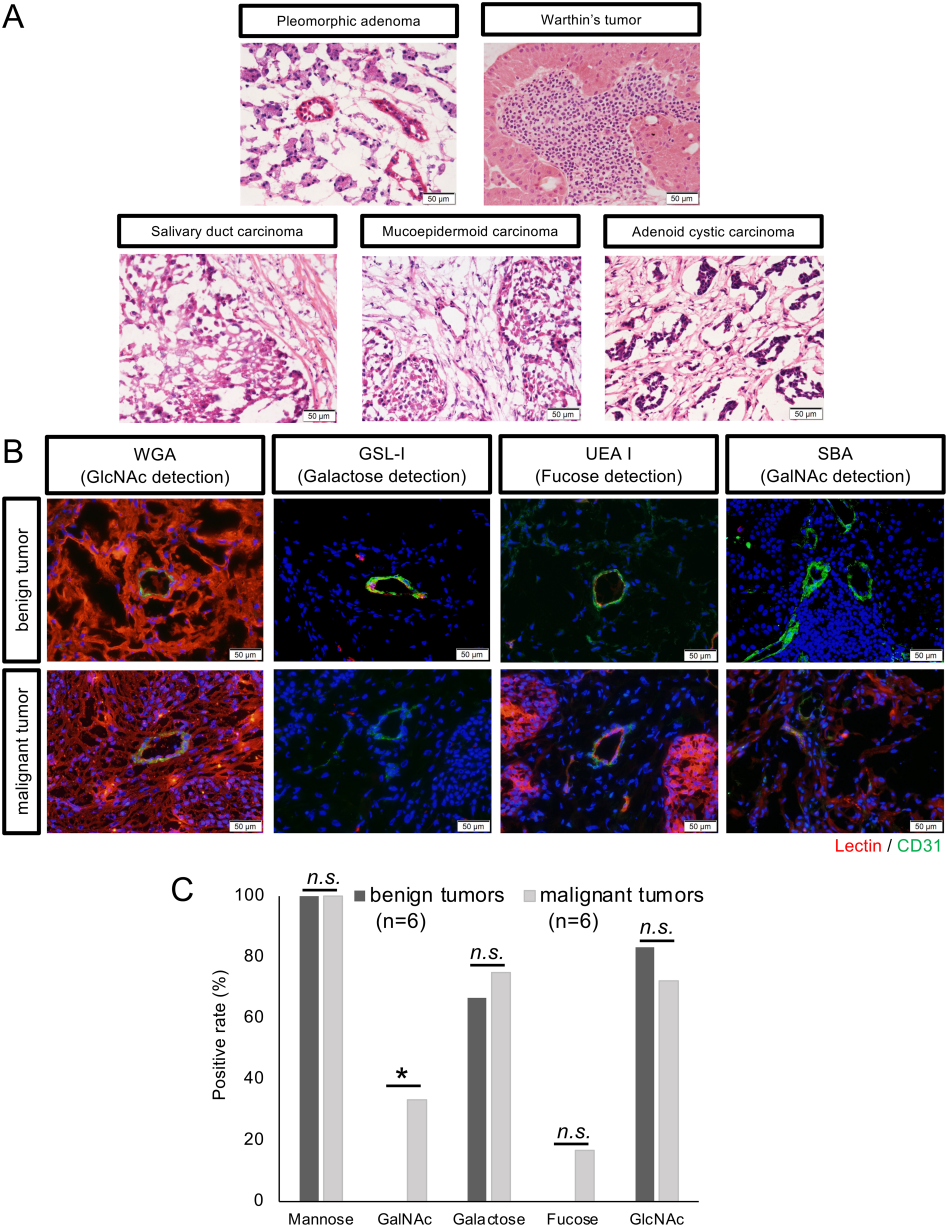
Differences in the staining patterns of benign and malignant tumors. (A) Representative hematoxylin–eosin (HE) staining of fresh frozen specimens in this study. (B) Some lectins showed positive staining in both benign and malignant tumors, negative staining in both tumors, and positive staining only in malignant tumors. Nuclei were stained with DAPI. (C) GalNAc and fucose were positive only in malignant tumors. *p < 0.05; n.s., not significant.

To investigate glycan expression in salivary gland tumors, lectin staining was performed on surgically resected human salivary gland tumors. We then analyzed PA and WT as representative benign salivary gland tumors. SDC, MEC, and ACC were analyzed as representative malignant salivary tumors (Figure 1A). All staining data are presented in Table 1. Typical staining patterns for lectins positive in both benign and malignant tumors (WGA), negative for both (GSL I), and positive only for malignant tumors (UEA I, SBA) are shown in Figure 1B. Lectins that detect mannose, galactose, and GlcNAc did not differ between the benign and malignant tumors. Notably, GalNAc and fucose were detected only in malignant tumors, suggesting their potential as malignant tumor cell surface markers (Figure 1C).

**Table 1:** Staining patterns in all samples

Kit	No	Lectin	PA1	PA2	PA3	PA4	WT1	WT2	SDC1	SDC2	MEC1	MEC2	ACC1	ACC2
I	1	Con A	+	+	+	+	+	+	+	+	+	+	+	+
	2	DBA	−	−	−	−	−	−	−	−	±	−	−	−
	3	PNA	±	+	+	+	−	−	±	+	−	−	±	+
	4	RCA I	+	+	+	+	+	+	+	+	+	+	+	+
	5	SBA	−	−	−	−	−	−	±	−	±	−	−	−
	6	UEA I	−	−	−	−	−	−	−	−	−	+	−	−
	7	WGA	+	+	+	+	+	+	+	+	+	+	+	+
II	1	GSL‐I	−	−	−	−	−	−	+	+	−	−	−	−
	2	PSA	+	±	±	±	+	+	+	+	+	+	+	+
	3	LCA	±	−	±	±	±	+	+	+	±	+	+	±
	4	PHA‐E	+	+	+	+	+	+	+	+	+	+	+	+
	5	PHA‐L	+	+	+	+	+	+	±	±	+	+	+	+
	6	WGA	−	−	−	−	−	−	−	−	−	−	−	−
III	1	DSL	+	+	+	±	+	+	+	±	+	+	±	+
	2	ECL	+	+	±	+	+	+	+	±	+	+	±	+
	3	GSL‐II	−	−	−	±	+	±	−	−	±	±	−	−
	4	Jacalin	+	+	+	±	±	+	±	±	±	±	+	+
	5	LEL	+	+	+	+	±	±	+	+	±	±	+	+
	6	STL	±	±	±	+	±	±	+	+	+	±	+	+
	7	VVL	−	−	−	−	−	−	+	+	±	−	−	−

With screening kit I, concanavalin A (Con A), *Ricinus communis* agglutinin (RCA) I, and wheat germ agglutinin (WGA) were positive in all tumors. *Dolichos biflorus* agglutinin (DBA) was weakly positive only for mucoepidermoid carcinoma (MEC)‐1, soybean agglutinin I (SBA) was weakly positive only for salivary duct carcinoma (SDC)‐1 and MEC1, and *Ulex europaeus* agglutinin (UEA) I was weakly positive only for MEC‐2. With screening kit II, *Pisum sativum* agglutinin (PSA), *Len culinaris* agglutinin (LCA), and *Phaseolus vulgaris* erythroagglutinin (PHA‐E) and leucoagglutinin (PHA‐L) were weakly positive‐to‐positive in most tumors, whereas WGA was negative in all tumors. *Griffonia simplicifolia* lectin (GSL) I was positive only in SDC‐1 and SDC‐2. With screening kit III, all tumors were weakly positive‐to‐positive for *Datura stramonium* lectin (DSL), *Erythrina cristagalli* lectin (ECL), jacalin, *Lycopersicon esculentum* lectin (LEL), and *Solanum tuberosum* lectin (STL). *Vicia villosa* lectin (VVL) was positive only for SDC‐1 and SDC‐2 and weakly positive for MEC‐1. UEA I was specifically positive for MEC, whereas GSL I and VVL were specifically positive for SDC.

PNA was positive in all PA cases but negative in all WT cases (supplementary material, Figure S1). GSL II was positive or weakly positive in all WT cases, whereas PA was negative in all but one case.

### VVL was negative in the vascular endothelial cells of SDC, and positive only in the SDC tumor cells

**Figure 2:**
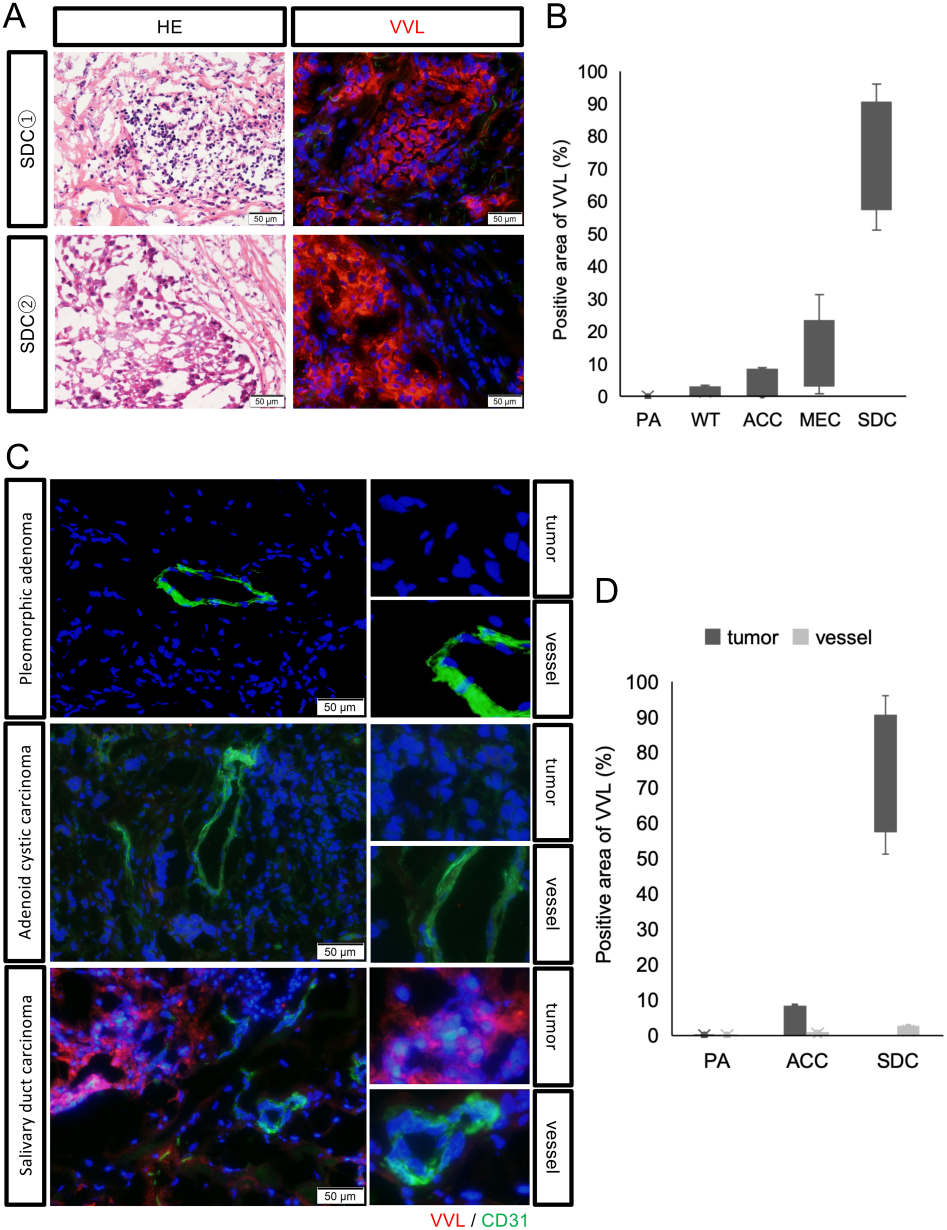
*Vicia villosa* lectin (VVL) only stains the tumor cells in salivary duct carcinoma (SDC). (A, B) VVL only stained malignant tumors, specifically SDC. CD31‐positive area indicates the vascular endothelium. (C, D) VVL did not stain the vascular endothelium but specifically stained the SDC tumor cells. Nuclei were stained with DAPI.

To investigate GalNAC expression in SDC tumors, VVL staining was performed. VVL was strongly stained in SDC (Figure 2A). VVL was more positive in malignant tumors than in benign tumors, particularly SDC (Figure 2). Glycocalyx is abundantly expressed in vascular endothelial cells; in some cases, it is not stained in vascular endothelial cells, but only in tumor cells. Notably, VVL was specifically positive in SDC tumor cells but negative in vascular endothelial cells in SDC tumor tissue, suggesting its mutually exclusive expression (Figure 2C,D). Comparing tumor cells and vascular endothelium in other tumor types, neither the vascular endothelium nor the SDC tumor cells were strongly stained.

### *GALNT7* is highly expressed in SDC

**Figure 3:**
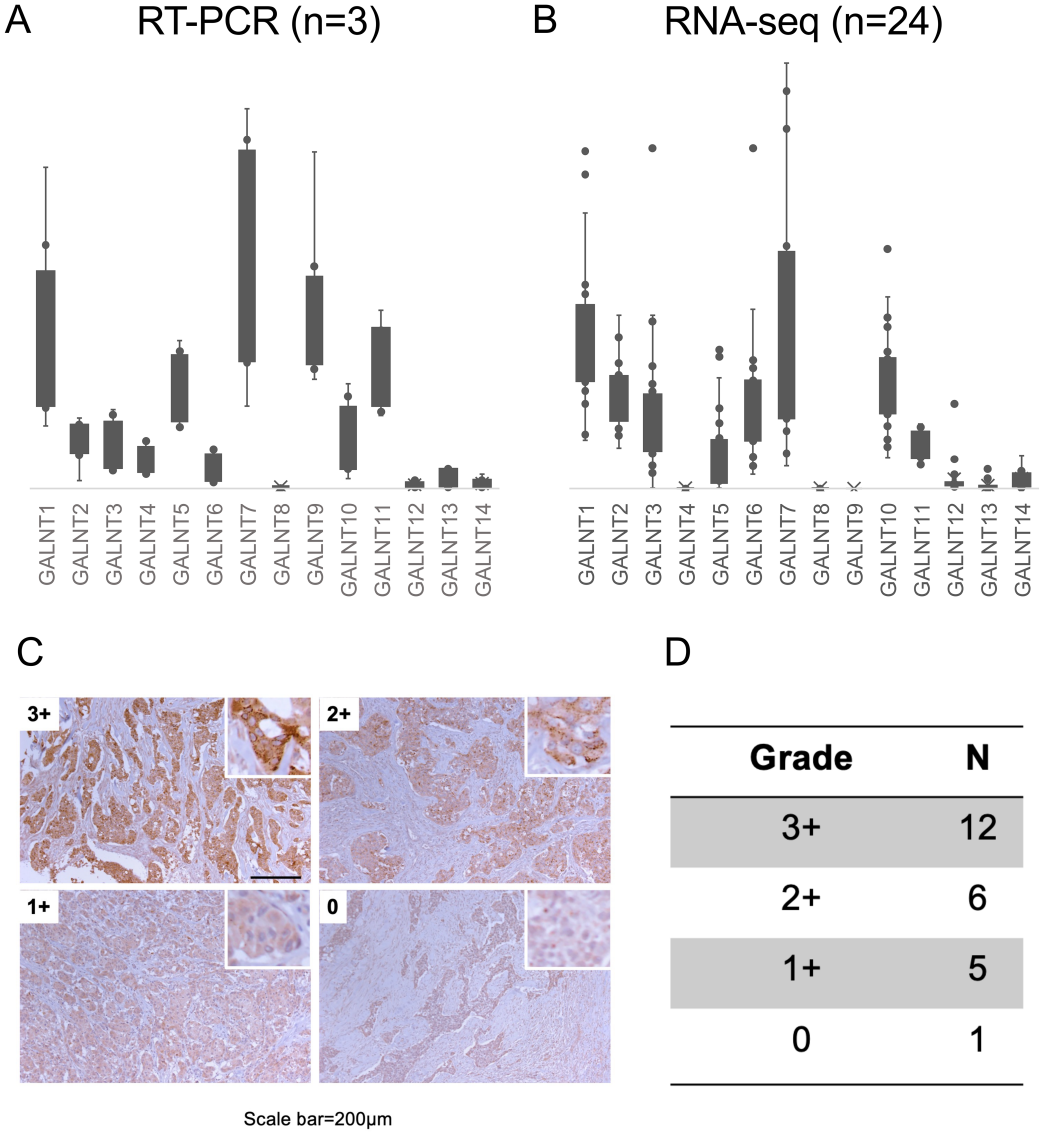
N‐acetylgalactosaminyltransferase (GALNT)‐7 is highly expressed in SDC. (A) Reverse transcription‐polymerase chain reaction (RT‐PCR) revealed that *GALNT7* was highly expressed in SDC. (B) Public RNA‐sequencing dataset showed similar results. (C) Representative images of GALNT7 immunohistochemical staining in SDC tissues, categorized by staining intensity (0, 1+, 2+, 3+). The insets show magnified views. Scale bar = 200 μm. (D) Quantification of GALNT7 staining intensity in 24 SDC cases. The table indicates the number of cases for each staining grade. Positive staining was defined as 1+, 2+, or 3+, while 0 indicated negative staining.

To analyze the GALNT subtype involved in O‐glycosylation in SDC, we performed RT‐PCR using three freshly frozen specimens. *GALNT7* was highly expressed in SDC (Figure 3A). To confirm the results, we analyzed a public RNA‐seq dataset of 24 SDC cases. Notably, similar results were obtained. These data indicate that *GALNT7* influences O‐glycosylation in SDC more than the other *GALNT* family genes (Figure 3B).

### GALNT7 is highly expressed at the protein level in SDC

To validate the high expression of GALNT7 at the protein level in SDC, we performed immunohistochemistry on 24 FFPE SDC samples using a GALNT7‐specific antibody. The staining intensity was graded on a scale of 0, 1+, 2+, and 3+, with 0 indicating negative staining and 1+, 2+, and 3+ indicating positive staining (Figure 3C). Out of 24 SDC cases, 12 cases (50%) showed strong positive (3+) staining, 6 cases (25%) showed moderate positive (2+) staining, 5 cases (20.8%) showed weak positive (1+) staining, and only 1 case (4.2%) showed negative (0) staining for GALNT7 (Figure 3D). These results demonstrate that GALNT7 is frequently and highly expressed at the protein level in SDC, consistent with our mRNA expression data. This finding further supports the notion that GALNT7 plays a significant role in O‐glycosylation and potentially in Tn‐MUC1 formation in SDC.

### VVL and Tn‐MUC1 staining is only positive in the tumor cells of SDC

**Figure 4:**
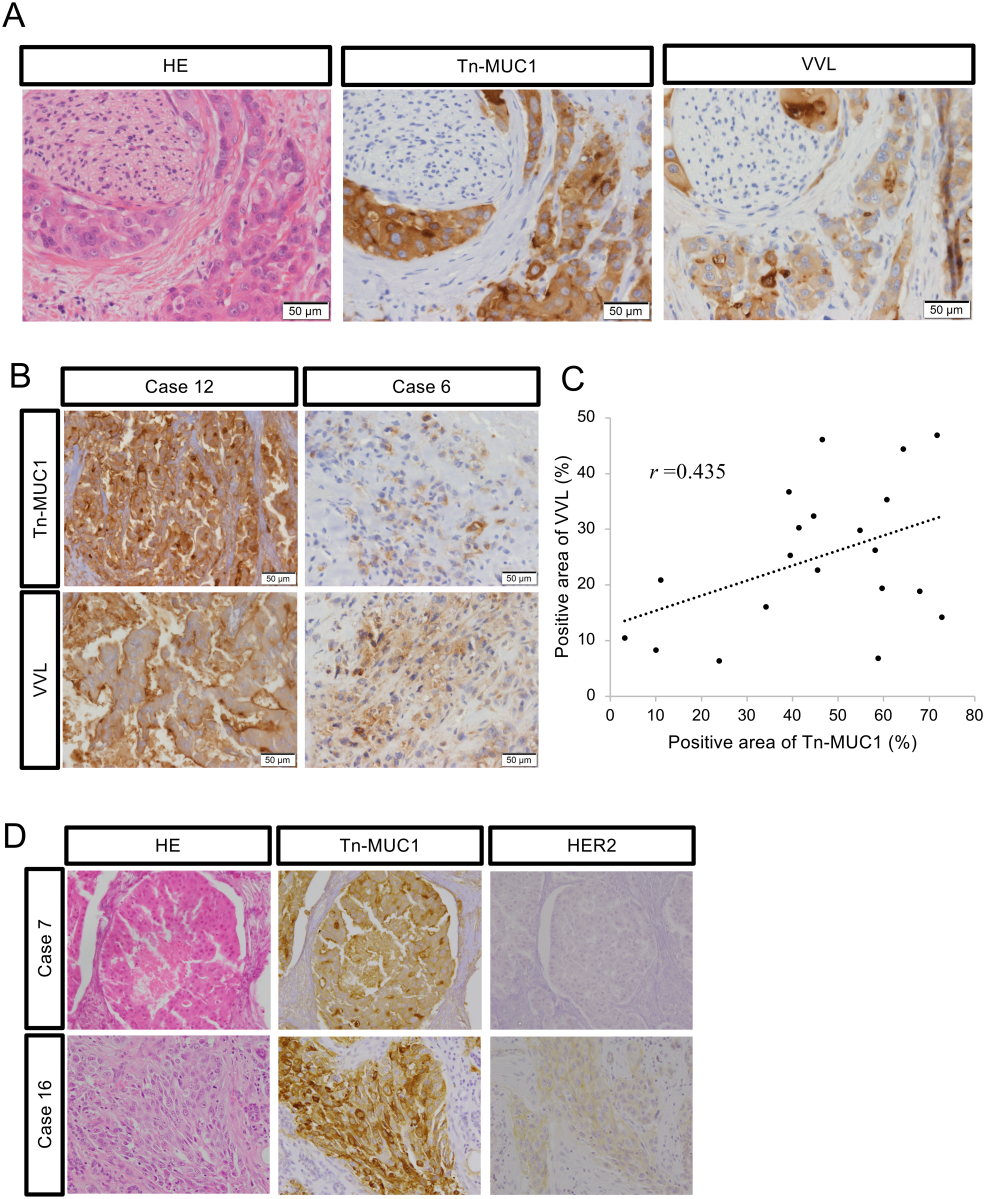
VVL and mucin 1 with Tn antigen (Tn‐MUC1) are only positive in SDC tumor cells. (A) Typical staining patterns for Tn‐MUC1 and VVL. (B) Case 12 was strongly positive, whereas case 6 was weakly positive for both Tn‐MUC1 and VVL. (C) Tn‐MUC1 and VVL correlate, with a correlation coefficient of 0.435. (D) Cases 7 and 16 were human epidermal growth factor receptor type 2 (HER2)‐negative but Tn‐MUC1‐positive.

Based on the fact that VVL lectin staining specifically recognizes Tn‐MUC1, Tn‐MUC1 and VVL lectin expression were evaluated using clinical specimens from 20 SDC cases. Patient characteristics are shown in Table 2. Notably, in all cases, Tn‐MUC1 and VVL were positive only in the same tumor regions. A typical example is presented in Figure 4A. VVL and Tn‐MUC1 were strongly positive, as in case 12, and weakly positive, as in case 6 (Figure 4B). The staining intensities of Tn‐MUC1 and VVL were moderately correlated (*r* = 0.435, *p* = 0.056) (Figure 4C). These data suggest that VVL expression is a surrogate of Tn‐MUC1 expression in SDC tumors.

**Table 2:** SDC patient characteristics

Case	Age	Sex	Primary	T	N	M	Initial treatment	Recurrence	Outcome
1	61	M	Parotid	4a	2b	0	Surgery	+	Death
2	63	M	Parotid	4	2b	0	Surgery	+	Death
3	77	F	Parotid	2	0	0	Surgery	−	Alive
4	62	M	Parotid	4a	2b	0	Surgery	−	Death
5	81	M	Parotid	4a	1	0	Surgery	+	Death
6	45	M	Submandibular	3	0	0	Surgery	+	Death
7	68	M	Oral cavity	4a	2b	0	Surgery	+	Alive
8	75	M	Parotid	4a	2b	0	Surgery	+	Death
9	64	M	Parotid	2	0	0	Surgery	+	Alive
10	84	M	Parotid	2	2b	0	Surgery	−	Alive
11	69	M	Parotid	3	2b	0	Surgery	−	Death
12	71	M	Parotid	4a	2b	0	Surgery	+	Alive
13	82	M	Submandibular	2	2b	0	Surgery	+	Death
14	62	M	Parotid	4a	2b	0	Surgery	+	Death
15	81	M	Parotid	2	0	0	Surgery	−	Alive
16	72	M	Parotid	2	2b	0	Surgery	−	Alive
17	75	M	Parotid	4a	2c	1	Chemotherapy	+	Alive
18	40	M	Parotid	1	0	0	Surgery	−	Alive
19	59	M	Parotid	3	2b	1	Chemotherapy	+	Death
20	68	M	Parotid	3	0	0	Surgery	−	Alive

A total of 20 cases are included. The mean age was 68 years, 95% were male, and 85% had parotid gland origin; 60% of cases had recurrence, and 50% died.

To investigate whether Tn‐MUC1 is a notable cell surface marker in the HER2‐negative SDC subgroup, HER2 immunohistochemical staining was performed and compared with Tn‐MUC1 expression. It is known that some SDC cases express HER2, which serves as a therapeutic target using an anti‐HER2 molecular targeted antibody (trastuzumab). In this study, 10 cases (50%) were HER2‐positive. In other words, Tn‐MUC1 may be positive even in HER2‐negative SDC (Figure 4D). Thus, Tn‐MUC1 may be a therapeutic target for HER2‐negative SDC.

### Correlation between Tn‐MUC1 and prognosis in SDC

**Figure 5:**
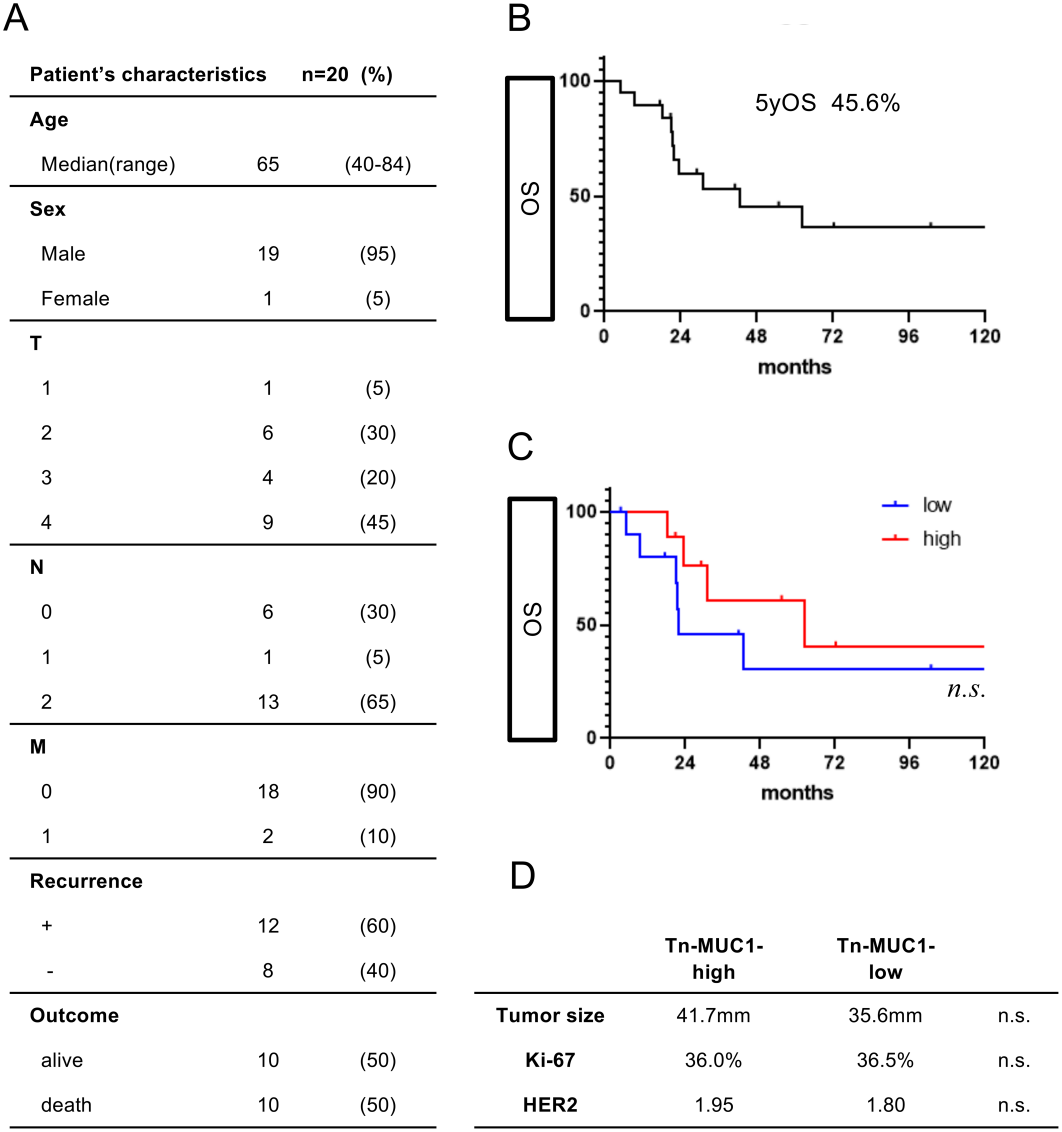
Correlations between Tn‐MUC1 and various clinical indicators. (A) Patient clinical characteristics. (B) Five‐year overall survival (OS) was 45.6%. (C) Tn‐MUC1 staining intensity was not associated with prognosis. Log‐rank test was used for the analysis; n.s., not significant. (D) Staining intensity of Tn‐MUC1 was not related to tumor size, proliferation (Ki‐67), or HER2 staining intensity; n.s., not significant.

We examined the prognosis of 20 patients with SDC treated at the Gifu University Hospital. Patient characteristics are shown in Figure 5A. The 5‐year overall survival (OS) was 45.6% (Figure 5B). In general, Tn‐MUC1 expression is associated with poor prognosis and high malignancy. However, in this study, Tn‐MUC1 staining intensity was not associated with the poor prognosis of SDC (Figure 5C). Furthermore, the staining intensity of Tn‐MUC1 was not related to tumor size, proliferation ability (Ki‐67), or HER2 staining intensity (Figure 5D).

## Discussion

Many reports on lectin staining for head and neck cancer were published in the 1980s and the 1990s, and most of these reports used FFPE specimens instead of freshly frozen specimens. However, formalin fixation destroys the glycan structure; therefore, it may not reflect the actual expression of glycans *in vivo*. To date, five reports have used any of the 20 types of lectins in salivary gland tumors [33, 34, 35, 36, 37], but none have been investigated as comprehensively as in this study. Yang *et al* reported that Con A, RCA, and WGA are positive in MEC [34], similar to the present study. In addition, Sobral *et al* reported that all histological grades of MEC are positive for Con A, whereas UEA I shows differences in staining intensity depending on the tumor grade [33].

The utility of lectins as potential cancer therapeutic tools and carrier proteins has been reported in various carcinomas [38, 39, 40, 41]. However, there is a problem with lectins as drug treatments; Con A is known to cause liver failure due to its toxicity. PHA‐L can also cause gastrointestinal symptoms, such as vomiting and diarrhea [42]. In this study, GSL I of SDC2, VVL of SDC1, and GSL II of MEC2 were stained only in tumor cells but not in vascular endothelial cells. However, further studies, including animal experiments, are needed to show that these lectins are safe carrier proteins.

This study also suggested that GALNT7 is involved in the formation of Tn‐MUC1 in SDC. It has been reported that GALNT7 affects prognosis in several cancers, including prostate cancer, breast cancer, and glioma [43, 44, 45]. GALNT7 is the only gene effector that controls the height of the glycocalyx, and upregulation of GALNT7 alters O‐glycosylation and promotes tumor growth [43]. This study also revealed that GALNT7 is the most highly expressed member of the GALNT family, and GALNT7 may be correlated with prognosis in SDC as in other cancers.

Tn‐MUC1 antibody used in this study was developed to recognize only the Tn antigen, a representative molecule of TA‐MUC1 [46, 47]. Previous studies have analyzed the expression of Tn‐MUC1 in various cancers, but none have explored its roles in salivary gland tumors [48]. Tn‐MUC1 is highly expressed in most cancers, accounting for 100% of stomach, 71% of colon, 75% of lung, 83% of breast, and 80% of ovarian cancers, although the number of such cases is small. In head and neck cancer, the expression of TA‐MUC1 in laryngeal cancer has been analyzed with a positivity rate of 54% [49].

Owing to its expression in many cancer types, various treatments targeting Tn‐MUC1, including chimeric antigen receptor‐T cells (CAR‐T) [50, 51, 52, 53] and vaccines, have been developed [54, 55, 56]. Furthermore, various antibody–drug conjugates (ADCs) such as ENHERTU® (trastuzumab deruxtecan) targeting HER2 in breast and gastric cancers have been developed [57, 58, 59]. ADCs are complex anticancer drugs consisting of an antibody, a binding substance called a linker, and a payload [60]. Currently, six ADCs have been approved by the European Medicines Agency and US Food and Drug Administration for the treatment of solid tumors, and many new ADCs including TA‐MUC1 are in clinical trials. Thus, Tn‐MUC1 has the potential to be a new therapeutic application for other cancers.

This study revealed that Tn‐MUC1 was expressed in SDC tumor cells, suggesting its potential as a new diagnostic marker for SDC. A limitation of this study is that salivary gland tumors are rare cancers, so the number of cases that can be analyzed in a single‐center study is limited. Therefore, it is possible that no correlation was observed between the staining intensity of Tn‐MUC1 and prognosis. Secondly, frozen samples are more suitable for accurate glycan analysis, but FFPE samples were used in this study. In the future, it is necessary to increase the number of cases by conducting prospective studies at multiple centers.

## Conclusion

To the best of our knowledge, this is the first report on the comprehensive glycan analysis of salivary gland tumors via lectin staining of fresh frozen sections. Lectins are useful for differentiating salivary gland tumors, and VVL was specifically expressed in tumor cells of SDC. Tn‐MUC1, which is formed by GalNAc specifically bound to VVL, was also highly expressed in SDC. In addition, it was suggested that GALNT7 is involved in the formation of Tn‐MUC1 in SDC. These results suggest that Tn‐MUC1 may be a novel potential marker for the diagnosis of SDC.

## Author contributions statement

MK, RK and HT conceived the study and performed the experiments. MK, RK, HS and HT analyzed the data and wrote the first draft of the manuscript. T Ohashi, TI, HO, AH and T Ogawa checked the data and edited the manuscript. All authors wrote the manuscript and provided final approval for the submitted and published versions of the manuscript.

## Supporting information


**Figure S1.** Salivary gland benign tumors can be differentiated using peanut agglutinin (PNA) and *Griffonia simplicifolia* lectin (GSL) II
**Table S1.** Lectin screening kits
**Table S2.** N‐acetylgalactosaminyltransferase (*GALNT*) primers

## Data Availability

RNA‐seq data were downloaded from the Gene Expression Omnibus database (https://www.ncbi.nlm.nih.gov/geo/; accession no. GSE138581). The data that support the findings of this study are available from the corresponding author (HS), upon reasonable request.
