# Supplementary material for: Targeting the cancer glycocalyx in salivary duct carcinoma: tumor‐associated mucin 1 (Tn‐MUC1) as a novel cell surface marker
Source: J Pathol Clin Res. 2025 Aug 22;11(5):e70042. doi: 10.1002/2056-4538.70042 (PMC12372492; doi:10.1002/2056-4538.70042)
Supplement: Supplementary file 1 — Figure S1. Salivary gland benign tumors can be differentiated using peanut agglutinin (PNA) and Griffonia simplicifolia lectin (GSL) II Table S1. Lectin screening kits Table S2. N‐acetylgalactosaminyltransferase (GALNT) primers [file CJP2-11-e70042-s001.pdf]

Targeting the cancer glycocalyx in salivary duct carcinoma: tumor-associated mucin 1 (Tn-MUC1) as a novel cell surface marker

M Kuroki, R Kawaura *et al*., *J Pathol Clin Res*, https://doi.org/10.1002/2056-4538.70042

Supplementary Figure S1

Supplementary Tables S1 and S2

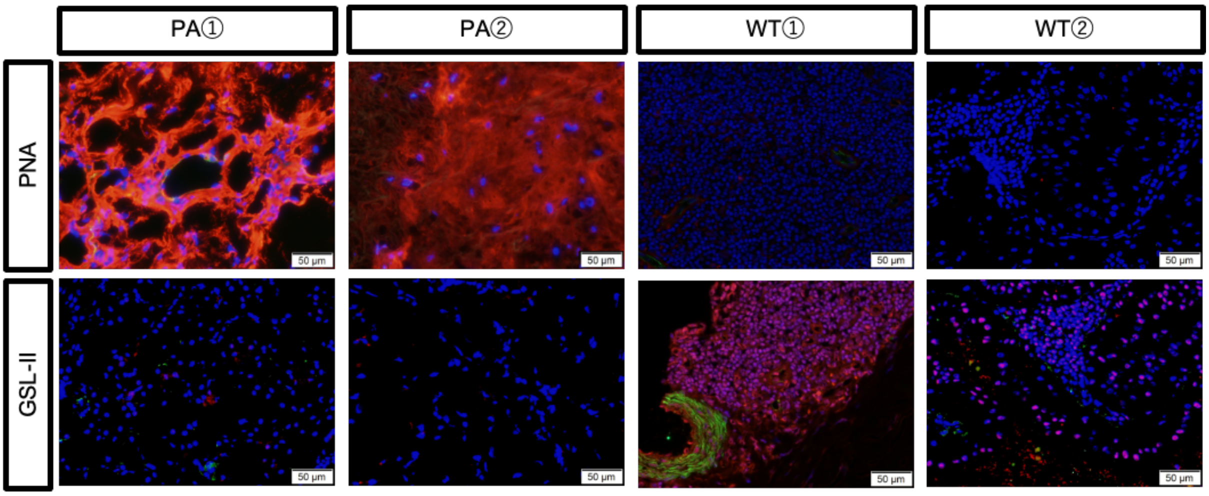

Figure S1. Salivary gland benign tumors can be differentiated using peanut agglutinin (PNA) and *Griffonia simplicifolia* lectin (GSL) II. PNA was positive in all pleomorphic adenoma (PA) cases but negative in all Warthin tumor (WT) cases. GSL II was positive in all WT cases but negative in 75% of PA cases.

Table S1. Lectin screening kits. Twenty types of lectins were used in this study. Screening kits I, II, and III included seven, six, and seven types of lectins, respectively. Glycans specifically bound to lectins.

Kit	No.	Lectin	Common abbreviation	Primary sugar specificity
I	1	Concanavalin A	Con A	Mannose
	2	*Dolichos biflorus* agglutinin	DBA	N-Acetylgalactosamine
	3	Peanut agglutinin	PNA	Galactose
	4	*Ricinus communis* agglutinin I	RCA I	Galactose, N-acetylgalactosamine
	5	Soybean agglutinin I	SBA	N-Acetylgalactosamine
	6	*Ulex europaeus* agglutinin I	UEA I	Fucose
	7	Wheat germ agglutinin	WGA	N-Acetylglucosamine
II	1	*Griffonia simplicifolia* lectin I	GSL-I	Galactose
	2	*Pisum sativum* agglutinin	PSA	Mannose
	3	*Len culinaris* lectin	LCA	Mannose
	4	*Phaselous vulgaris* Erythroagglutinin	PHA-E	Complex structures
	5	*Phaselous vulgaris* leucoagglutinin	PHA-L	Complex structures
	6	Wheat germ agglutinin, succinylated	Succinylated WGA	N-Acetylglucosamine
III	1	*Datura stramonium* lectin	DSL	N-Acetylglucosamine
	2	*Erythria cristagalli* lectin	ECL	Galactose
	3	*Griffonia simpliciforia* lectin II	GSL-II	N-Acetylglucosamine
	4	Jacalin	Jacalin	Galactose
	5	*Lycopersicon esulentum* lectin	LEL	N-Acetylglucosamine
	6	*Solanum tuberosum* lectin	STL	N-Acetylglucosamine
	7	*Vicia villosa* lectin	VVL	N-Acetylgalactosamine

Table S2. N-acetylgalactosaminyltransferase (*GALNT*) primers. *GALNT* primers were designed using Primer-BLAST.

Gene name	Forward primer (5' → 3')	Reverse primer (5' → 3')
GALNT1	CACCTCCTTGATTTGGGTACTC	GGAATGACGACTGGTTTCCC
GALNT2	ACCAGGTGGAGAGTGATAAGC	TCCTCAGGATCATTGCTGTAGTC
GALNT3	CACCTGCAATACTGCTGAAGG	ACAGAGGTTCTAGCCAACCAT
GALNT4	ACAGTGGCCTATATCTTCGTGG	CTCCTGCGGAGGCATGAAAA
GALNT5	GGGCGAGTCTTGGCATTTATC	CCCGAGTGTTGATCTCACTGAAT
GALNT6	ACAGCGTCCTACACACCAC	CTTCTCCTTTAGGTGCTCCTCT
GALNT7	TCCTCGGTAACTTTGAACCCA	GCGGTCCAGTGAGATCATGTC
GALNT8	GACACGCGAGACTACAGATGT	GATGGCCCGTTGTATAATGGAC
GALNT9	GAAGCCCTACAACAACGACA	GGGTTCGACATGGGGATGTTC
GALNT10	TCAGCGCGTAGGAAATGGAG	GGAGAGAGCGATTCAAGGAGA
GALNT11	TTCGGTATTTCTGTTATGGGTGC	ACGACTGTCTATCACATCGTCAA
GALNT12	GTGCGGCTGCACCAGATTA	AACTGTCCGAAGGAGAGTTGA
GALNT13	TTGCCCTTAATAGAAGTCTGCCA	TGGGGAACGATTTATCACACTG
GALNT14	CACTGCTGGTGTATTGCACG	CGGATCAGATGCGTAGGGG
